# Evaluation of personal protection afforded by repellent-treated sandals against mosquito bites in south-eastern Tanzania

**DOI:** 10.1186/s12936-020-03215-7

**Published:** 2020-04-08

**Authors:** Onyango P. Sangoro, Tegemeo Gavana, Marceline Finda, Winfrida Mponzi, Emmanuel Hape, Alex Limwagu, Nicodem J. Govella, Prosper Chaki, Fredros O. Okumu

**Affiliations:** 1 Environmental Health and Ecological Sciences, Ifakara Health Institute, Ifakara, Tanzania; 2 International Centre for Insect Physiology and Ecology, Nairobi, Kenya; 3 Institute of Biodiversity, Animal Health and Comparative Medicine, University of Glasgow, Glasgow, UK; 4 Pan African Mosquito Control Association, Nairobi, Kenya; 5 School of Public Health, Faculty of Health Sciences, University of the Witwatersrand, Johannesburg, South Africa; 6 School of Life Sciences and Bio Engineering, The Nelson Mandela, African Institution of Science and Technology, Tengeru, Arusha, United Republic of Tanzania

**Keywords:** Residual malaria transmission, New tools, Transfluthrin, Transfluthrin-treated footwear, Vector borne diseases, Ifakara

## Abstract

**Background:**

Outdoor and early evening mosquito biting needs to be addressed if malaria elimination is to be achieved. While indoor-targeted interventions, such as insecticide-treated nets and indoor residual spraying, remain essential, complementary approaches that tackle persisting outdoor transmission are urgently required to maximize the impact. Major malaria vectors principally bite human hosts around the feet and ankles. Consequently, this study investigated whether sandals treated with efficacious spatial repellents can protect against outdoor biting mosquitoes.

**Methodology:**

Sandals affixed with hessian bands measuring 48 cm^2^ treated with 0.06 g, 0.10 g and 0.15 g of transfluthrin were tested in large cage semi-field and full field experiments. Sandals affixed with hessian bands measuring 240 cm^2^ and treated with 0.10 g and 0.15 g of transfluthrin were also tested semi field experiments. Human landing catches (HLC) were used to assess reduction in biting exposure by comparing proportions of mosquitoes landing on volunteers wearing treated and untreated sandals. Sandals were tested against insectary reared *Anopheles arabiensis* mosquitoes in semi-field experiments and against wild mosquito species in rural Tanzania.

**Results:**

In semi-field tests, sandals fitted with hessian bands measuring 48 cm^2^ and treated with 0.15 g, 0.10 g and 0.06 g transfluthrin reduced mosquito landings by 45.9%, (95% confidence interval (C.I.) 28–59%), 61.1% (48–71%), and 25.9% (9–40%), respectively compared to untreated sandals. Sandals fitted with hessian bands measuring 240 cm^2^ and treated with 0.15 g and 0.10 g transfluthrin reduced mosquito landings by 59% (43–71%) and 64% (48–74%), respectively. In field experiments, sandals fitted with hessian bands measuring 48 cm^2^ and treated with 0.15 g transfluthrin reduced mosquito landings by 70% (60–76%) against *Anopheles gambiae* sensu lato, and 66.0% (59–71%) against all mosquito species combined.

**Conclusion:**

Transfluthrin-treated sandals conferred significant protection against mosquito bites in semi-field and field settings. Further evaluation is recommended for this tool as a potential complementary intervention against malaria. This intervention could be particularly useful for protecting against outdoor exposure to mosquito bites. Additional studies are necessary to optimize treatment techniques and substrates, establish safety profiles and determine epidemiological impact in different settings.

## Background

Mosquito control using long-lasting insecticidal nets (LLINs) and indoor residual spraying (IRS) has had a substantial impact on malaria transmission globally [1–3]. However, deliberate scale up of LLINs and IRS has led to the emergence of behaviourally resilient malaria vectors [4] that evade these tools by increasingly feeding and resting outdoors [5–8]. These changes, associated with the suppression of the once predominant local vector [4–9] attenuate the impact of LLINs and IRS [4]. This shift in mosquito species composition and consequently to mosquito behaviour that define the biological limits of LLINs and IRS, coupled with practices that expose human hosts to outdoor mosquito biting [10, 11] have resulted in persistent malaria transmission outdoors (residual transmission) [12, 13].

With the increasing significance of outdoor malaria transmission [12], there is need to develop and deploy tools that control mosquito bites outdoors [14]. There are several strategies that are being developed that can be used to tackle transmission outdoors. Killing adult mosquitoes when they feed upon sugar using attractive toxic sugar baits (ATSB) [15–21], when they feed on livestock that have been sprayed with or ingested endectocides [22, 23], use of odour-baited mosquito landing boxes outdoors [24–26], use of topical repellents in the early evenings [27, 28] and larval source management [29, 30].

In addition to the above tools, spatial repellents are also being proposed as supplementary to LLINs [31, 32]. Spatial repellents are insecticidal products that act in the vapour phase to prevent human-vector contact by causing mosquitoes to move away from the source of chemical stimulus, interferes with the vector response to stimuli or otherwise causes feeding inhibition [33, 34]. There are several formats through which spatial repellents are dispensed, such as mosquito coils, vaporizer mats and liquid vaporizers [24, 35, 36]. Recently, an alternative emanator delivery format for volatilizing transfluthrin at ambient temperatures have proven efficacious against mosquito bites [37–41]. Although effective, these emanator formats require that the host is confined to a protected air space, therefore, limiting mobility [38–40]. In order to impact outdoor biting, spatial repellents delivery formats must be optimized to protect users wherever they are outdoors [42].

It has previously been reported that the malaria vector, *Anopheles gambiae* sensu lato (s.l.) prefers to bite humans on the lower limbs and that this behaviour is mediated by convection currents arising off the host [43, 44]. A recent study demonstrated that the highest densities of bites from these vectors occur on host body parts that are closest to the ground [45], and that protecting these body parts results in significant reduction in mosquito bites to the host [45, 46].

Exploiting this mosquito behaviour, this study assessed the impact of integrating spatial repellents into sandals on mosquito bites outdoors. In addition to reducing mosquito bites overall on the human host [45, 46], treating footwear with long-lasting spatial repellents also presents an opportunity to overcome concerns of frequent reapplication, which is often encountered when using topical repellents [27], making it prohibitively expensive. Using footwear as a delivery format of spatial repellents will also overcome the challenge of limited mobility of recently developed emanator formats, that require the host to be within the treated air space of stationary emanators [37–39]. Integrating insecticide into footwear that is locally made, low cost and worn ubiquitously across communities on a daily basis will likely promote uptake and the attendant effectiveness as it does not require any change in human behaviour [27, 47, 48].

## Methods

### Study area and facilities

**Fig. 1:**
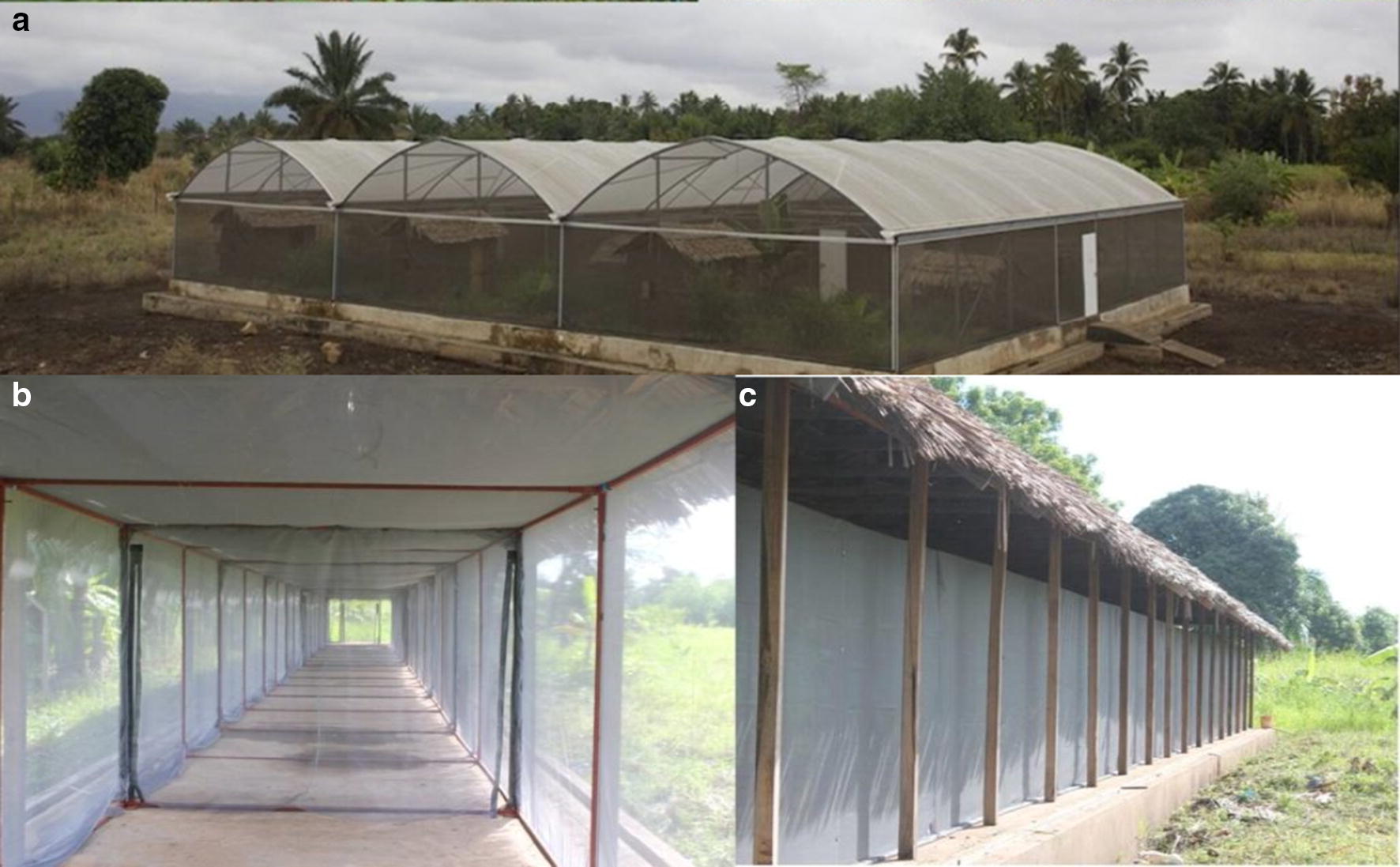
Pictorial illustration of (IHI) semi field cages, **a** large multi-compartment system; **b** inside sections of the long, tunnel-shaped screened semi-field system; **c** outside view of the long tunnel-shaped screened semi-field system

Large cage semi-field evaluations were conducted at the Ifakara Health Institute’s (IHI) experimental station, in Kining’ina village (8.11° S, 36.67° E), approximately 6 km north of Ifakara town, in Kilombero district, south-eastern Tanzania. Experiments were conducted inside two different types of large screened cages. The first was a large multi-compartment system covering 553 m^2^ in ground area, and 4.5 m in height (Fig. 1a) [49–51]. The experiments were conducted in two compartments inside this large multi-compartment system each covering 36 m^2^ in ground area, and 4.5 m in height. The second cage was a long, tunnel shaped screened system measuring 110 m long × 2 m wide × 2.5 m high (Fig. 1b, c) [37].

Field experiments were conducted in villages in Kilombero and Ulanga districts, in south-Eastern Tanzania. These study areas experience a wet season between March and June and a dry season in August to October, with a mean annual rainfall of 1200–1400 mm and a daily temperature range of 20–32 °C [52]. Malaria is endemic and current transmission is primarily mediated by *Anopheles funestus* sensu stricto (s.s.) and *Anopheles arabiensis.* The main malaria control intervention used in the study area is LLINs. Pyrethroid resistance is prevalent in both *An. arabiensis* [53], and *An. funestus* [54].

### Mosquitoes

For the semi-field evaluations, laboratory-reared, pyrethroid susceptible *An. arabiensis* (Ifakara strain) were used in the semi-field experiments. Larvae were fed on Tetramin^®^ fish food and maintained at temperatures of 28–29 °C. Pupae were placed in a separate room in emergence bowls inside a 30 × 30 × 30 cm netting cage and a 10% glucose solution provided for the emergent adults. Temperatures were maintained at 27 ± 3 °C and relative humidity at 70–90%. The insectary was maintained at a 12:12 (light:dark) photoperiod. The mosquitoes used in experiments were 4–9 days old nulliparous females. The mosquitoes were starved for 6 h before each experiment.

### Volunteers

Adult male volunteers (18 to 40 years old) participated in the experiments. The volunteers were trained on objectives, benefits and potential risks of the study and recruited only if they provided written informed consent. All volunteers were highly experienced in the procedure of human landing catch, wherein mosquitoes attempting to bite a volunteer’s legs are captured immediately upon landing [55]. The volunteers were instructed not to use any fragranced soap or perfume, tobacco or alcohol throughout the experiment period.

### Transfluthrin-treated footwear prototype

**Fig. 2:**
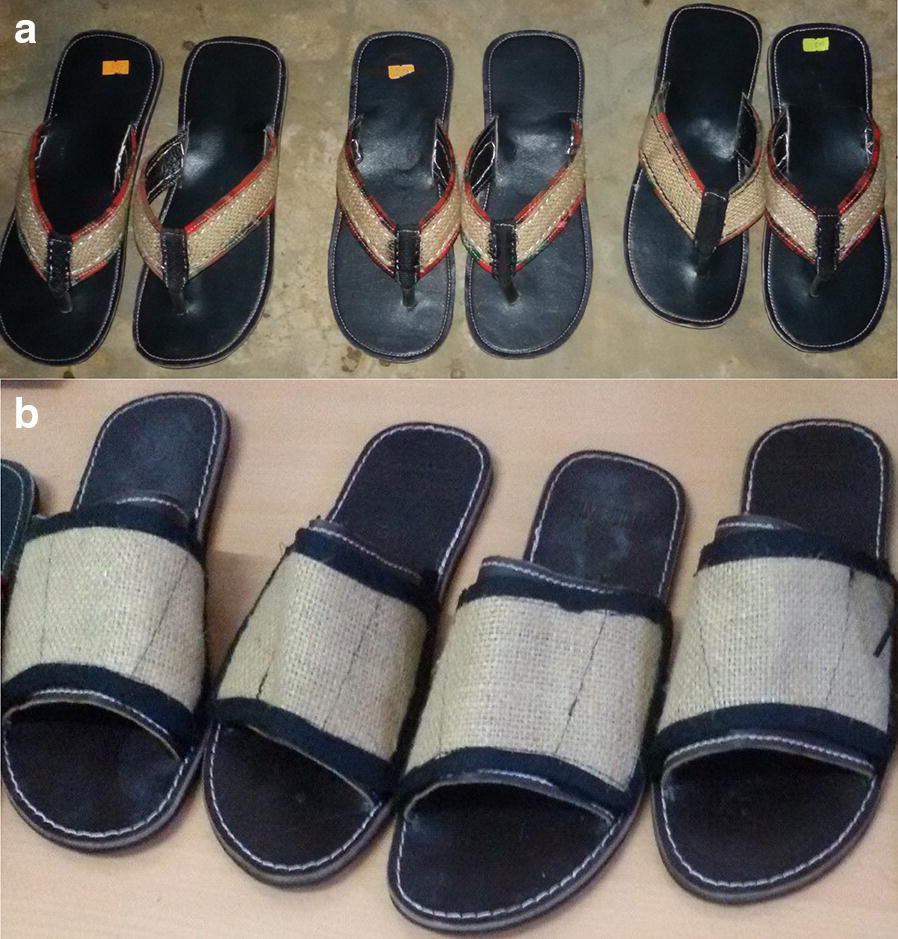
Prototype designs of the transfluthrin-treated sandals; **a** design using hessian fabric measuring 240 cm^2^ and **b** design with hessian fabric measuring 48 cm^2^

Locally manufactured leather sandals were fitted with transfluthrin-treated hessian fabric affixed onto the straps of the sandals. The bands were fitted on the top leather surface of the sandal straps, so that there was no direct contact with the volunteer’s skin (Fig. 2). Hessian fabric was used because it is readily available in Tanzania and has an optimal adsorbent capacity [37]. Transfluthrin, a pyrethroid insecticide recommended by the World Health Organization (WHO) for control of flying insects, such as mosquitoes and flies [56], was selected as spatial repellent. Transfluthrin is a highly volatile pyrethroid with a vapour pressure of 9 × 10^−4^ Pa at 20 °C [56]. This property makes it suitable for use in tropical regions and at low-cost because no additional heating is required to evaporate the chemical [57]. In addition, previous studies carried out at Ifakara Health Institute have demonstrated efficacy of transfluthrin-impregnated hessian fabric against mosquito bites in semi-field and field experiments [37–40] further affirming the potential of transfluthrin-treated hessian substrates in malaria control.

The technical grade transfluthrin used to treat the hessian fabric was donated by SC Johnson (Racine, Wisconsin, USA). Identical sandals treated with axion detergent and water were used as negative controls.

### Experiment 1: Semi-field experiments to determine a dose–response relationship for transfluthrin in terms of protection against mosquito bites

Three different amounts of 97% technical grade transfluthrin were used to treat the hessian bands attached to the sandals in the first set of experiments; 0.06 g, 0.10 g and 0.15 g transfluthrin was mixed with 94 ml, 90 ml and 64 ml of Axion^®^ liquid detergent (Orbit Chemical Industries Ltd, Kenya), respectively, to enable solubility in water [37]. This emulsion was then mixed with 100 ml of water for both 0.06 g and 0.10 g transfluthrin and 74 ml of water for 0.15 g transfluthrin. These amounts were used to ensure there was no left-over emulsion which might undermine the treatment dose.

Rectangular hessian fabric bands measuring 48 cm^2^ were dipped in the resulting emulsion containing either of the three amounts of transfluthrin and soaked until complete saturation and all the emulsion had been absorbed after which they were suspended at ambient temperatures indoors, protected from direct sun exposure, and allowed to dry for 24 h. Three pairs of sandals were then fitted with the treated hessian fabric bands, each containing either of the three amounts of transfluthrin (Fig. 2b). A pair of matching negative controls for each treatment were prepared in the same way, except the treatments were done with only Axion^®^ liquid detergent and water without any transfluthrin.

Two volunteers were asked to wear knee-length shorts to standardize the area of the lower limbs exposed. They sat on low chairs in experimental compartments measuring 6 × 6 m inside the semi-field facility (Fig. 1a). One compartment of the semi-field system was used for testing the treatment while the other tested the control sandal. The experiments were conducted one at a time starting with the sandal fitted with the hessian fabric that had the lowest amount of transfluthrin. Each night, 200 female *An. arabiensis* mosquitoes were released at the centre of each experimental compartment at 18:00 h, with the volunteer sitting approximately 10 m from each other. The volunteers collected all mosquitoes attempting to bite the exposed lower limbs for 45 min of each hour and rested for 15 min. Mosquito collections were done from 18:00 to 06:00 h the next morning. Each volunteer was given a head torch and siphon for aspirating the mosquitoes. The mosquitoes were kept in separate paper cups for each hour of collection. At the end of each night of experiment, the recaptured mosquitoes were killed using petroleum ether, counted and recorded for each hour.

The experiments were conducted in a binary cross-over design, where each amount of transfluthrin (sandal pair) was tested for 8 consecutive nights against the control. Only one sandal pair, containing either of the three treatment amounts was tested per night. On the first night of each experiment testing the different transfluthrin doses, the treatment and control sandals were randomized to the experimental compartments. Volunteers were then rotated each following night between the two experimental compartments of the semi-field system. The experimental compartments were separated by a similar-sized chamber in between that acted as a buffer in the event of spillover effects of the treatment. The treatment and control sandals remained in the same compartment throughout the experiments to minimize the potential impact of residual effect of transfluthrin.

### Experiment 2: Semi-field experiments to evaluate the of impact of hessian fabric surface areas on mosquito bites

Another set of experiments was conducted to assess the impact of surface area of treated hessian fabric on mosquito bites. Hessian fabric pieces measuring 48 cm^2^ and 240 cm^2^ were each treated with 0.10 g and 0.15 g of transfluthrin using the same methodology described above, affixed onto sandals and their efficacies against mosquito bites evaluated.

The same binary cross-over design as experiment one above was used, and the experiments were replicated 8 times (nights) for each treatment.

### Experiment 3: Experimental field evaluation of the efficacy of transfluthrin-treated sandals against bites from wild mosquitoes

A binary crossover design, similar to the semi-field experiments was used in the two study villages. The 48 cm^2^ hessian fabric pieces treated with 0.15 g transfluthrin were evaluated in these experiments as they demonstrated significant efficacy against mosquitoes. The field tests were conducted by two volunteers who carried out the experiments over 12 nights in each study village. The tests were conducted outdoors, next to rice fields and away from human dwellings, and these sites, each with two positions, were fixed throughout the experiments. The experiments were conducted on separate nights in the two villages.

The volunteers, wearing transfluthrin-treated sandals sat at a randomly selected position approximately 20 m away from the volunteer wearing the untreated sandal. The volunteers collected all mosquitoes attempting to bite the exposed lower limbs for 45 min of each hour and rested for 15 min from 18:00 to 06:00 h. The volunteers were blinded to the treatment status of the sandals. At the end of the hourly collections, the paper cups holding the mosquitoes were placed in a cool box until the next morning when the mosquitoes were killed using petroleum ether. The mosquitoes in each paper cup were counted by each volunteer and the numbers recorded. The mosquitoes were sorted into anophelines and culicines. Each anopheline was stored in a 5 ml micro-centrifuge tube (Eppendorf^®^ tubes) containing silica gel and later morphologically identified by experienced entomologists. All specimen of *An. gambiae* s.l. caught were assumed to be *An. arabiensis,* since contemporaneous molecular analysis of mosquitoes from the same villages have consistently confirmed these to be of *An. arabiensis* [28, 54, 58, 59].

Other mosquito genera caught were stored in batches of five per micro-centrifuge tube and later identified by experienced entomologists [60].

### Experiment 4: Assessment of whether transfluthrin-treated sandals divert host-seeking mosquitoes to persons not wearing the sandals

This experiment was carried out to determine whether the use of transfluthrin-treated sandals would put nearby non-users at a greater risk of being bitten by diverting mosquitoes to non-users. In this experiment, a volunteer wearing transfluthrin-treated sandals sat at one end of an experimental compartment while another volunteer wearing untreated sandals sat at the other end of the same compartment, approximately 10 m away. This was the treatment compartment. Four hundred laboratory-reared female *An. arabiensis* mosquitoes were released from a small cage midway between the two volunteers. The number of mosquitoes attempting to bite the volunteers were caught, throughout the night. Another pair of volunteers sitting in a comparative compartment (control compartment), replicated this experiment using untreated sandals. To establish diversion, the number of mosquitoes caught by the volunteer wearing the untreated sandals in the treatment compartment were compared to the number of the mosquitoes caught by the volunteers in the comparative compartment to determine if the volunteer who sat next to transfluthrin-treated sandals caught more mosquitoes compared to either volunteer in the comparative compartment with untreated sandals only. Similar to the semi-field experiments above, the sandals remained in the same position in each respective chamber and only the volunteers rotated between positions to control for individual attractiveness. The limitations of this design have been outlined in the limitations section of the manuscript.

### Experiment 5: Semi-field experiments to assess the efficacy of transfluthrin-treated sandals on laboratory-reared *Anopheles* and *Aedes* mosquitoes

**Fig. 3:**
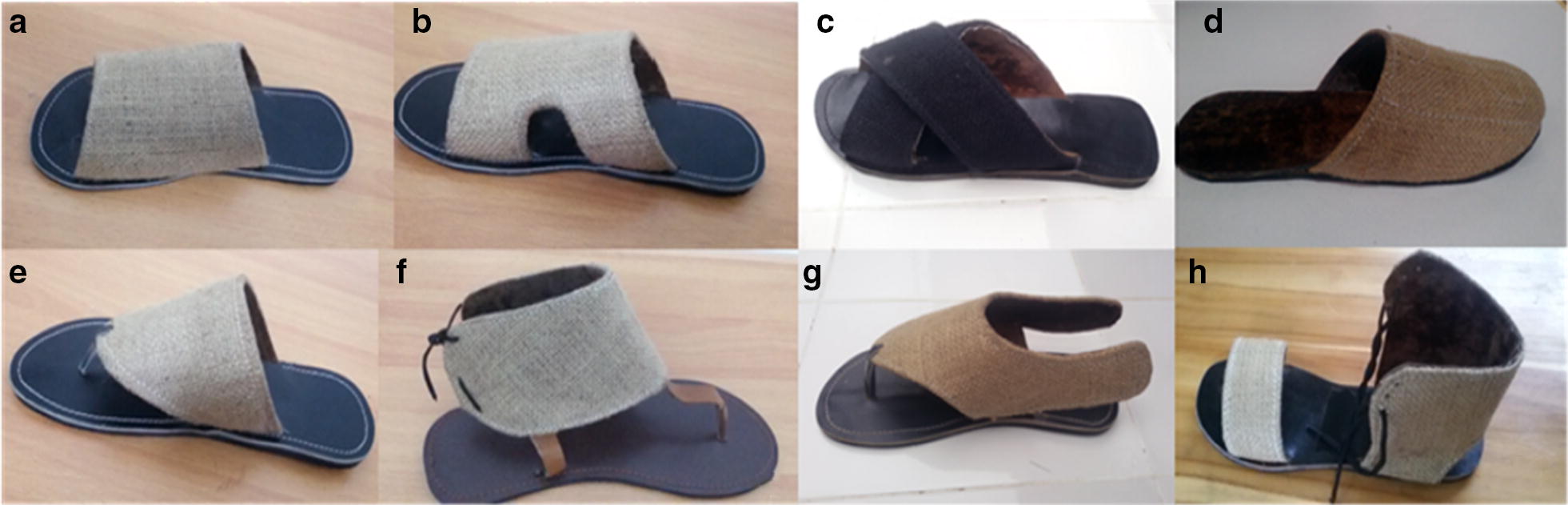
Prototypes of transfluthrin-treated sandal designs developed; **a** Male sandal design 1. **b** Male sandal design 2. **c** Male sandal design 3. **d** Male sandal design 4. **e** Female sandal design 1. **f** Female sandal design 2. **g** Female sandal design 3. **h** Female sandal design 4

Eight different sandal prototypes (Fig. 3), treated with 0.05 g of transfluthrin were later developed and tested in the semi field system against the following laboratory reared mosquito species; A (male design 1)—*An. arabiensis* and *Aedes aegypti*; B (male design 2)—*An. arabiensis*; C (male design 3)—*An. arabiensis* and *Ae. aegypti*; D (male design 4)—*An. arabiensis*; E (female design 1)—*An. gambiae* s.s. and *Ae. aegypti*; F (female design 2)—*An. arabiensis* and *Ae. aegypti*; G (female design 3)—*An. arabiensis* and *Ae. aegypti* and H (female design 4)—*An. arabiensis*. All sandals developed could not be tested against all mosquito species because of logistical and cost implications. Different prototype sandals were therefore tested against randomly selected mosquito species. This was done to select the sandal prototype that offered the best protection against the respective randomly selected mosquito species. The results from these experiments were later pooled to evaluate the impact of transfluthrin-treated sandals on different mosquito species.

The surface areas of the hessian fabric used for the different sandal designs were as follows; A—395 cm^2^; B—327 cm^2^; C—330 cm^2^; D—≈ 400 cm^2^; E—346 cm^2^; F—780 cm^2^; G—640 cm^2^, and H—325 cm^2^. All the designs developed were treated with 0.05 g of 97% technical grade transfluthrin (Shenzhen Sunrising Industry Company, China) following risk assessments of transfluthrin-treated sandals. The treatments followed the same methodology described above.

A binary cross-over design similar to the previous experiments was used to evaluate the impact of the sandals against their respective randomly chosen mosquito species. The experiments were replicated for 6 days for each sandal design. In experiments where *Anopheles* species were used, experiments were conducted from 18:00 to 00:00 h. (midnight). When *Aedes* species were used, experiments were conducted from 06:00 to 1200 h. as they are day-biting vectors.

One hundred mosquitoes were released in two batches of 50 mosquitoes after every 3 h in each experimental compartment. The volunteers were asked to wear a customized clothing that covered their whole body but left their lower limbs exposed by pulling the lower parts of their overalls to their knees. Mosquitoes were released into the two experimental compartments at the same time and left to acclimatize for 15 min before the volunteers entered their respective compartments assigned randomly before the start of the experiments. At the end of the hourly collections, the paper cups holding the mosquitoes were placed in a cool box until the next morning when the mosquitoes were killed using 70% ethanol. Field technicians counted and the sorted the mosquitoes into anophelines and culicines. Each anopheline was stored in a 5 ml micro-centrifuge tube (Eppendorf tubes) containing silica gel and later taken to the laboratory for further species identification. Other mosquito genera caught were stored in batches of five per micro-centrifuge tube and later identified by experienced entomologists.

### Experiment 6: Field experiments to assess the efficacy of sandals treated with transfluthrin on wild mosquito bites

The field experiments for the two prototype designs of sandals were conducted in Minepa and Lupiro villages in Ulanga district, south-Eastern Tanzania. However, in these experiments, instead of HLCs, the mosquito electrocuting trap (MET) [61, 62] was used to test to the efficacy of transfluthrin-treated sandals against wild mosquitoes.

Similar to the semi-field experiments, a binary cross over design was used when assessing the efficacy of the transfluthrin-treated sandals using the MET in the field. Two METs were placed at least 20 m apart, next to rice fields and away from human dwellings. The volunteers wearing transfluthrin-treated or untreated sandals were randomly assigned to their positions only on the 1st day of the experiments and on subsequent nights rotated between the two fixed trapping points. The volunteers sat with their lower limbs exposed inside the MET from 18:00 to 00:00 h for the first 45 min of each hour and collected electrocuted mosquitoes during the last 15 min of every hour. These experiments were conducted for a total of 12 days, 6 days for each prototype. These two prototypes (male and female sandals design 4) were selected because they provided the best protection against mosquito bites in semi field experiments above.

### Statistical analysis

Data from both the semi-field and field experiments were recorded in a spreadsheet showing date of data collection; name of volunteer; whether the volunteer wore treated or untreated sandals; position of volunteer and the number of mosquitoes caught in each hour.

The effect of transfluthrin-treated sandals on the risk of exposure to mosquito bites was quantified by fitting a generalized linear mixed effects model with a negative binomial distribution to account for the over dispersion of mosquito count data. To account for day to day variation, date was included in the model as a random effect. The treatments on the sandals were included in the model as independent variables and the number of mosquitoes caught of those released as the dependent variable. Variations associated with fluctuations in temperature, humidity, wind direction and speed were assumed to be captured by the date random effect. Date, together with volunteers and hour and were treated as random effects.

To assess whether treated sandals divert mosquito to non-users, the number of mosquitoes caught by the untreated sandals in the two experimental compartments were compared. Diversion was modelled using three different values; the untreated sandal that was set up in the same compartment as the treated sandal compared with the two untreated sandals that were used in the comparative compartment. The model was used to derive incidence rate ratios (IRR) for numbers of biting mosquitoes in each of the three scenarios; untreated sandals in the treatment compartment and the two untreated sandals used in the comparative compartment.

## Results

**Fig. 4:**
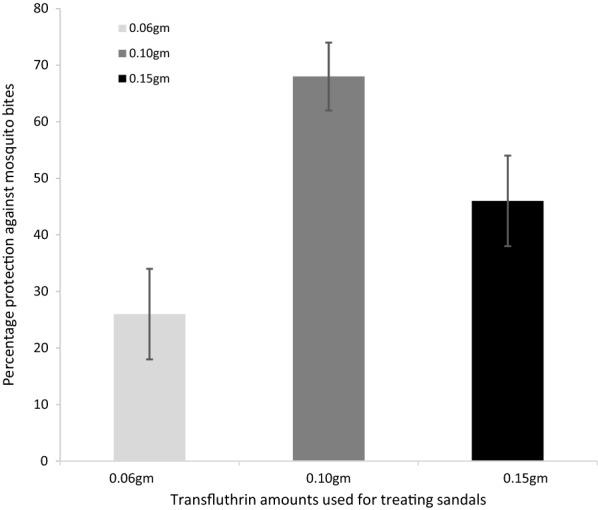
Graph showing the protection conferred by sandals treated with different amounts of transfluthrin against *An. arabiensis* mosquito bites in semi field experiments

Sandals affixed with hessian fabrics measuring 48 cm^2^ and treated with 0.15 g, 0.10 g and 0.06 g transfluthrin reduced mosquito landings by 45.9%, (95% confidence interval (C.I.) 28–59%), 61.1% (48–71%) and 25.9% (9–40%), respectively, when compared to untreated sandals in semi-field experiments (Fig. 4).

**Fig. 5:**
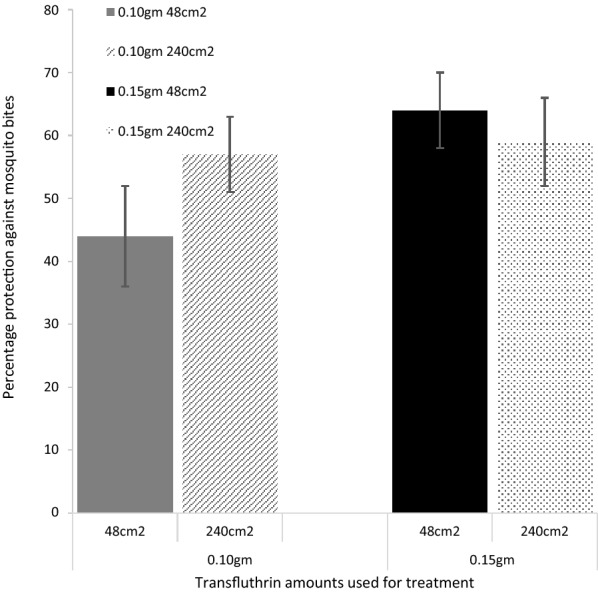
Graph assessing the impact of the surface area of transfluthrin-treated hessian fabric on protection against laboratory reared *An. arabiensis* mosquito bites in semi field experiments

In the second set of experiments, hessian fabrics measuring 48 cm^2^ and 240 cm^2^ and each treated with both 0.10 g and 0.15 g of transfluthrin were compared to determine the impact of surface area on mosquito bites. Sandals affixed with hessian fabric measuring 240 cm^2^ and treated with 0.15 g and 0.10 g transfluthrin reduced mosquito landings by 59% (43–71%) and 64% (48–74%), respectively. Sandals affixed with hessian fabric measuring 48 cm^2^ hessian fabric and treated with 0.15 g and 0.10 g transfluthrin reduced mosquito landings by 57% (43–67%) and 44% (26–56%), respectively (Fig. 5).

In the third experiment, sandals affixed with hessian fabrics measuring 48 cm^2^ and treated with 0.15 g transfluthrin were tested against wild mosquitoes in two villages in south-Eastern Tanzania. Transfluthrin-treated sandals significantly reduced all mosquito species landing by 65.6% (95% C.I. 59–71%) compared to the untreated sandals. Against *An. gambiae* s.l., these sandals reduced mosquito landings by 70.75% (58.2–75.7%) (Table 1).

**Table 1:** Field evaluation of efficacy of transfluthrin-treated sandals against pooled mosquitoes and *Anopheles gambiae* s.l.

Field experiment	Incidence rate ratio IRR (95% C.I.)	p-value	z-score	Mean number of mosquitoes (standard deviation)	
				Treatment	Control
All mosquito species	0.34 (0.29–0.41)	< 0.0001	− 12.33	31.25 (29.64)	90.64 (70.11)
*An. gambiae* s.l.	0.29 (0.24–0.40)	< 0.0001	− 7.87	1.79 (2.73)	5.28 (4.99)

In the fourth experiment, diversion of transfluthrin-treated sandals was assessed. In the experimental compartment the volunteer wearing transfluthrin-treated sandals caught an average of 4.81 (Standard deviation, S.D., 8.32) mosquitoes while the volunteer wearing untreated sandals caught an average of 14.46 (S.D., 24.08) mosquitoes per night. In the comparative compartment, where both volunteers wore untreated sandals, the first volunteer caught an average of 16.25 (S.D., 31.06) mosquitoes while the second volunteer caught an average of 15.34 (S.D., 29.75) mosquitoes per night. Volunteers sitting next to a user of transfluthrin-treated sandal did not receive significantly more mosquito bites when compared to volunteers sitting next to non-users of transfluthrin-treated footwear (Table 2).

**Table 2:** Assessment of whether transfluthrin-treated sandals divert host-seeking mosquitoes to persons wearing untreated sandals

Treatment groups	Incidence rate ratio IRR^a^ (95% C.I.)	p-value	z-score	Mean number of mosquitoes (standard deviation)	
				Treatment	Control
Repellent-treated sandal (experimental compartment)	0.32 (0.23–0.44)	< 0.0001	− 6.81	4.81 (8.23)	14.46 (24.08)
Untreated sandal 1 (comparative compartment)	0.89 (0.67–1.18)	0.42	− 0.81	15.37 (29.75)	14.46 (24.08)
Untreated sandal 2 (comparative compartment)	0.80 (0.59–1.07)	0.13	− 1.53	16.25 (31.01)	14.46 (24.08)

^a^The reference sandal was the untreated sandal in the experimental chamber

The fifth experiment evaluated the efficacy of sandals treated with 0.05 g of transfluthrin against laboratory reared mosquito landings in large cage semi-field experiments. Transfluthrin treated sandals significantly reduced *An. arabiensis* landings by 55% (95% C.I. 48.2–60.9%) when compared to untreated sandals. When tested against *Ae. aegypti*, treated sandals reduced mosquito landings by 37.2% (24.4–47.9%). Against *An. gambiae* s.s. transfluthrin-treated sandals reduced mosquito landings by 60.3% (47.4–70%) (Table 3).

**Table 3:** Semi-field experiments to assess the efficacy of sandals treated with 0.05 g of transfluthrin against different species of laboratory reared mosquito bites

Mosquito species	Incidence rate ratio IRR (95% C.I.)	p-value	z-score	Mean number of mosquitoes (standard deviation)	
				Treatment	Control
*An. arabiensis*	0.45 (0.39–0.52)	< 0.0001	− 11.09	4.05 (3.77)	8.95 (6.48)
*Ae. aegypti*	0.62 (0.52–0.75)	< 0.0001	− 4.90	7.20 (5.97)	11.71 (10.89)
*An. gambiae* s.s.	0.39 (0.30–0.53)	< 0.0001	− 6.46	3.94 (3.29)	9.56 (5.56)

In the sixth set of experiments, the efficacy of sandals treated with 0.05 g of transfluthrin was evaluated against wild mosquito species in the field. Transfluthrin-treated sandals reduced *An. gambiae* s.l. landings by 50% (95% C.I. 21.4–68.2%) and *Culex* spp. landings by 41% (23.8–53.7%) (Table 4).

**Table 4:** Field experiments to assess the efficacy of sandals treated with 0.05 g of transfluthrin against different species of wild mosquitoes using the Mosquito electrocuting trap (MET)

Mosquito species	Incidence rate ratio IRR (95% C.I.)	p-value	z-score	Mean number of mosquitoes (standard deviation)	
				Treatment	Control
*An. gambiae* s.l.	0.50 (0.32–0.79)	0.003	− 2.98	3 (4.73)	5.63 (8.08)
*Culex* spp.	0.59 (0.46–0.76)	< 0.0001	− 4.16	2.56 (3.04)	4.11 (3.71)

## Discussion

Use of transfluthrin-treated sandals reduced exposure to *An. gambiae* s.l. landings by at least 50% and 40% against *Culex* mosquitoes. Transfluthrin-treated sandals also provided useful protection against *Ae. aegypti* mosquitoes. This study adds to the mounting body of evidence that these tools (transfluthrin-impregnated substrates) can provide personal protection against different species of mosquito species and the growing arsenal of vector control tools that are currently being developed to supplement LLINs and IRS in the context of outdoor mosquito exposure [37–39, 41].

While the increase in transfluthrin amounts used for treatment may appear to be associated with increase in protection, there was no significant difference in reduction of mosquito landings when using either 0.10 g or 0.15 g transfluthrin. This suggests that increasing the amounts of spatial repellent used for treatment of sandals above a certain amount may not necessarily provide additional protection against mosquito bites. This finding is consistent with a previous study where 2 ml and 10 ml dose of transfluthrin were found to have no difference in bites reductions [40]. This may imply that like topical repellents, there might be a plateau in the protection provided by higher concentrations of transfluthrin repellents [41, 63].This study did not find any impact of treated surface areas on protection efficacy against mosquitoes. However, previous studies using treated hessian ribbons of about 2 m long provided greater protection efficacy against mosquito bites compared to the findings of this study [38–40]. The lack of impact of surface area of hessian substrate on mosquito landings observed in this study may mean that the differences in the surface areas of the substrates used in this study may not have been large enough to demonstrate an observable effect. These observations indicate that surface area of treated substrates might be an important factor to consider when developing these types of interventions.

Similar to other studies that exploited the potential use of transfluthrin-impregnated substrates as mosquito control tools, this study did not demonstrate any diversion of mosquito bites to non-users of transfluthrin-treated sandals [40]. Consequently, potential employment of transfluthrin-treated sandals is unlikely to place non-users at a greater risk of mosquito bites. This is one advantage that this intervention possesses over topical repellents [64, 65].

In addition to the aspect of diversion, transfluthrin-treated sandals also overcome the issue of daily compliance and reapplication that is faced with topical repellents and other spatial emanators, such as mosquito coils, as footwear is something that is already used ubiquitously in the community. This tool will, therefore, not require users to change their behaviour [66], a feature that will facilitate its acceptability and by extension effectiveness [48]. As a result, use of footwear may result in quicker uptake and extensive coverage which will likely make this tool effective in its implementation and scale up. More importantly is that repellent-treated sandals can be used outdoors and during the day where the effect of LLINs and IRS is attenuated [67], offering a complementary tool to tackle this niche of residual transmission and drive towards the goal of malaria elimination.

Despite the potential presented by repellent-treated sandals as a malaria control tool, it should be noted that these results were obtained from a single study. To realize the potential of this tool, there is need to conduct detailed additional studies to optimize the dose and formulations used in treatment of substrates and determine the release rates of the repellents into the airspace. There will also be need for public engagement and education of the efficacy, safe disposal techniques, retreatment and safety profiles of this technology.

Even though experiments to determine the longevity of protection provided by transfluthrin-treated sandals were not carried out, other studies have demonstrated the efficacy of transfluthrin-treated hessian substrates to last longer than 6 months [37–40]. Improved treatment/impregnation or encapsulation methods of the active ingredient are necessary to ensure safe optimal concentrations. In addition, the optimal substrates and surface areas to be treated/impregnated should be further explored to in order to establish the longevity of protection conferred by this tool.

One limitation of this study was that the treatments were not rotated between the experimental compartments in the semi-field experiments and the impact of the treated sandals may have been as a result of the experimental compartments and not the treatment itself. However, this outcome is unlikely as this bias was controlled for during the field experiments where the treatments were rotated between the experimental positions and the treated sandals still demonstrated significant reduction in mosquito landings.

Another limitation is that this study did not determine the longevity of protection, the area of protection ‘bubble’ provided and the impact under different environmental and epidemiological backgrounds of transfluthrin-treated sandals.

A third limitation is that mosquitoes were collected from only the lower limbs of the volunteers. It is possible that mosquitoes did bite other parts of the body that were not measured. Further studies need to be conducted to test the impact of the treated sandals on the whole human body.

## Conclusion

Transfluthrin-treated sandal provides a potential tool that could be used to attack residual malaria transmission that is mediated by early and outdoor biting *An. arabiensis* s.l. However, further studies will be required to optimize this tool before it can be deployed for testing under epidemiological conditions. Cost–benefit analysis as well as acceptability studies will also need to be conducted to determine the impact of this tool.

## Data Availability

The datasets used and/or analysed during the current study are available from the corresponding author on reasonable request.
